# Patients With Multiple Myeloma Have a Disbalanced Whole Blood Thrombin Generation Profile

**DOI:** 10.3389/fcvm.2022.919495

**Published:** 2022-06-27

**Authors:** Li Li, Mark Roest, Yaqiu Sang, Jasper A. Remijn, Rob Fijnheer, Karel Smit, Dana Huskens, Jun Wan, Bas de Laat, Joke Konings

**Affiliations:** ^1^Department of Platelet Pathophysiology, Synapse Research Institute, Maastricht, Netherlands; ^2^Department of Biochemistry, Cardiovascular Research Institute Maastricht (CARIM), Maastricht University, Maastricht, Netherlands; ^3^Department of Clinical Chemistry, Meander Medical Center, Amersfoort, Netherlands; ^4^Department of Internal Medicine, Meander Medical Center, Amersfoort, Netherlands; ^5^Department of Functional Coagulation, Synapse Research Institute, Maastricht, Netherlands

**Keywords:** blood cells, multiple myeloma, platelet function, thrombin generation, thrombosis

## Abstract

**Background:**

Multiple myeloma (MM) is associated with a high prevalence of bleeding and an increased risk of thrombo-embolism. MM patients have reduced platelet- and red blood cell (RBC) numbers in blood, which may indicate that the paradoxical hemostasis profile is a consequence of a disturbed platelet and RBC homeostasis.

**Objectives:**

To get better insight in the disbalanced hemostasis of MM patients.

**Methods:**

We conducted a case-control study on the whole blood (WB) coagulation profiles of 21 MM patients and 21 controls. We measured thrombin generation (TG) in WB and platelet poor plasma (PPP) of MM patients and controls.

**Results:**

In WB-TG, we observed that the median time to the thrombin Peak was 52% longer in MM patients than in controls, while the median endogenous thrombin potential until the Peak (ETPp) was 39% higher in MM-patients than in controls. In line with these findings, the levels of platelets, RBCs, white blood cells and agonist induced platelet activation were decreased in MM patients compared to controls. The plasma TG experiments showed no differences between MM-patients and controls.

**Conclusion:**

Patients with MM have a disturbed blood cell metabolism and a disbalanced WB-TG profile. This disbalance may explain the paradoxically high prevalence of bleeding symptoms in MM patients vs. an increased thrombosis risk. There was no disturbance observed in plasma TG, indicating that blood cells are the major determinants for the disbalanced hemostasis in MM patients.

## Introduction

Multiple myeloma (MM) is a hematological malignancy of the plasma cells, which is characterized by the production of abnormal monoclonal immunoglobulins, called M-protein. Patients with MM have an up to 28-fold increased risk of venous thromboembolism (VTE) and a high prevalence of bleeding complications (1). Although bleeding complications, such as nosebleeds, are common in MM, life-threatening hemorrhage is rare (2). The real cause of the high VTE risk in MM patients remains unclear, despite several studies on the relation between global coagulation and VTE risk (1, 3, 4). Platelet and red blood cell (RBC) levels of MM patients are reduced, and platelet function is disturbed. This disbalance may lead to both a pro- or an anti-coagulant phenotype, while intensive treatment of MM-patients with anti-platelet therapy, anti-coagulants, chemotherapy, immune modulating drugs (IMiD) and corticosteroids can further modify the thrombosis risk (5).

Common complications in patients with MM are anemia and thrombocytopenia (6, 7). This may disturb the coagulation in MM patients, because activated platelets and RBCs provide the physiological surface for the assembly of coagulation factors and coagulation factor complexes, including tenase and prothrombinase (8–10). Besides high prevalence of thrombocytopenia, platelets of MM patients are more frequently chronically activated during disease progression (11). Pro- or anti-coagulant changes in blood cells may contribute to the increased VTE risk in MM patients and the high prevalence of bleeding complications, and therefore it is important to investigate these cells in combination with coagulation. Therefore, we recently optimized a method to measure thrombin generation (TG) in whole blood (WB-TG) (12). TG is a global coagulation assay that measures the global capacity of blood plasma to form thrombin. Several clinical studies have shown that increased TG in platelet poor plasma (PPP) predicts an increased risk of (recurrent) VTE (13–15). The strength of the new WB-TG assay is that it includes the contribution of platelets, white blood cells and RBCs in the TG measurements (12).

This is the first study that investigates WB-TG coagulation profiles of MM patients and the effects of medication, with regard to a representative control group.

## Materials and Methods

### Study Population

In total, 21 patients with MM were recruited at the Meander Medical Center, Amersfoort, the Netherlands. Patients were included if they were previously diagnosed with MM, were patients at the Meander Medical Center, had a stable condition, and received treatment. Patients were excluded if they were pregnant, had a known coagulation defect, an active infection, bleeding, or other systemic diseases. The control group (*n* = 21) consists of the partner or a close friend with a comparable age and gender distribution, which donated blood at the same time and location as the patient. Controls were excluded if they had an active malignancy, a personal history of bleeding or thrombosis, were pregnant, had a known coagulation defect, an active infection, bleeding, or other systemic diseases. The study was approved by the Medical Ethical Committee of Maastricht University Medical Center, and patients and volunteers gave full written informed consent according to the Helsinki declaration.

### Reagents

Recombinant tissue factor (TF; Innovin^®^) was purchased from Siemens healthineers, Marburg, Germany. The glycoprotein VI (GPVI) agonist, collagen-related peptide (CRP-XL) was purchased from the University of Cambridge, UK. The fluorogenic substrate Z-Gly-Gly-Arg-aminomethylcoumarin (ZGGR-AMC) was purchased from Bachem (Basel, Switzerland). The calibrator (α2-macroglobulin-thrombin complex) and HEPES buffers containing 5 mg/ml or 60 mg/ml bovine serum albumin (BSA5 and BSA60) were prepared as described by Hemker et al. (16, 17). Synthetic phospholipids (PL) were obtained from Avanti Polar Lipids Inc. (Alabama, USA). MeSADP was purchased from Tocris Biosciences (Bristol, UK), the protease activated receptor (PAR)-1 agonist thrombin receptor activator peptide (TRAP-6 (SFLLRN) was from Bachem (Basel, Switzerland). The monoclonal antibodies used were FITC-conjugated PAC1, directed against the activated αIIbβ3 receptor, PE-conjugated anti-P-selectin (CD62P, clone AK4), APC-conjugated anti-GPIb (CD42b, clone HIP1), APC-conjugated anti-CD14 (monocyte marker) and PE-conjugated anti-αIIbβ3 (CD41a, clone HIP8), all purchased from BD Pharmingen (NJ, USA). HEPES-buffered saline (HBS, 10 mmol/l HEPES, 150 mmol/l NaCl, 1 mmol/l MgSO_4_, 5 mmol/l KCL, pH 7.4) and fixation solution (137 mmol/l NaCl, 2.7 mmol/l KCl, 1.12 mmol/l NaH_2_PO_4_, 1.15 mmol/l KH_2_PO_4_, 10.2 mmol/l Na_2_HPO_4_, 4 mmol/l EDTA, 0.5% formaldehyde) were prepared.

### Blood Collection and Plasma Preparation

Peripheral venous blood from patients and controls was collected aseptically by antecubital puncture via a 21-gauge needle into two 3.2% (109 mM) trisodium citrate vacuum tubes and one K2EDTA (7.2 mg) tube (BD Vacutainer System/Greiner). Citrated blood was used for whole blood experiments and EDTA blood was used for measuring complete blood cell count measured with Sysmex XN-9000 (Sysmex, Germany). All blood samples were kept at room temperature (RT) and used within 4 h after collection. PPP was prepared by double centrifugation of citrated WB at 2,840 g for 10 min. Plasma was aliquoted and frozen at −80°C before analysis.

### Whole Blood Thrombin Generation Measurement

WB-TG measurements were performed as described with modifications (12). In short, citrated WB was mixed with the substrate solution (ZGGR-AMC dissolved in BSA60). Subsequently, a solution containing trigger (TF) and CaCl_2_ was added to the WB and mixed. The volume ratio of WB, substrate solution, and trigger-containing solution was 3:1:2. Of the resulting mixture, 65 μL per well was transferred into 96-well plates (Corning, type number 2595). The final concentrations in the well were 50% WB, 0 or 1 pmol/L TF, 16.7 mmol/L CaCl_2_, and 416.7 μmol/L ZGGR-AMC. Each blood sample was calibrated by replacing the trigger-containing solution with a calibrator (corresponding with 300 nmol/L thrombin activity). Fluorescence signals were recorded with a Fluoroskan Ascent microplate fluorometer (Thermolabsystems) with λex = 355 nm and λem = 460 nm using Fluoroskan Ascent Software (version 2.6) at 37°C with an interval time of 6 seconds in triplicate. A dedicated preprogrammed spreadsheet template was used to calculate the WB-thrombogram parameters (lag time (minutes), time-to-peak (TTP, minutes), peak thrombin (peak, nmol/L), and endogenous thrombin potential until the peak (ETPp; nmol x min/L) from the experimental fluorescence data (12).

### Platelet Poor Plasma Thrombin Generation Measurement

TG in PPP was measured as described previously, using the calibrated automated thrombinography (CAT) method (18). In short, 80 μl of plasma was added to 20 μl of trigger-solution containing TF and PL. The reaction was started by dispensing 20 μl of substrate solution to the wells. Final concentrations were: 0, 1 or 5 pmol/L TF, 4 μM PL, 16.7 mmol/L CaCl2, and 416.7 μmol/L ZGGR-AMC. Each PPP sample was calibrated by replacing the trigger-solution with a calibrator (corresponding with 600 nmol/L thrombin activity). Data were acquired by specialized software from Thrombinoscope (Maastricht, the Netherlands). Resulting parameters include endogenous thrombin potential (ETP, area under the curve), peak thrombin (peak), lag time (time to thrombin generation initiation) and time-to-peak (TTP, time to reach the peak concentration of thrombin. In each run, normal pooled plasma (NPP) was measured on the same plate. The ETP and peak values of the study subjects were normalized as the percentage of the ETP and peak of the NPP tested in the same run, respectively. The preparation of NPP has been described previously (19). NPP consisted of PPP of 121 apparently healthy individuals (60 males, 61 females) with a median age of 30 (IQR: 25 – 43).

### Whole Blood Platelet Activation Test

Platelet activation tests for flow cytometric analysis were prepared as published earlier (20). Test conditions were no agonist, 30 μmol/L TRAP, 0.5 μg/ml CRP-XL, 5 μg/ml CRP-XL and 2 μmol/l MeSADP. Samples were analyzed on an Accuri C6 flow cytometer (BD Biosciences, USA). Median fluorescent intensity (MFI) in the FITC gate and PE gate was selected to determine activated αIIbβ3 (FITC-conjugated PAC1) or P-selectin density (PE-conjugated anti-P-selectin), respectively. The results of the whole blood platelet activation test are missing for 1 patient and 1 control.

### Measurement of Platelet Monocyte Complexes

Whole blood (20 μL) was incubated for 15 min with 20 μL reaction mixture consisting of monoclonal antibodies against monocyte CD14 and platelet αIIbβ3. Subsequently, BD FACSTM Lysing solution (Becton-Dickinson, diluted 10-fold with milliQ) was added, vortexed and incubated for 10 min at room temperature before analysis on an Accuri C6 flow cytometer. Gating was performed as described previously (21).

### Statistics

Statistical analyses were performed with SPSS version 25 and graphs were generated using GraphPad Prism software version 6. Normality of the data was assessed using the Shapiro-Wilk test. Data are represented as median with interquartile range (IQR; 25–75%). Comparisons between independent groups were performed with the Mann-Whitney U test. The Spearman test was used for correlation analysis. A two-sided *P*-value < 0.05 was considered statistically significant. Based on the following assumptions: alpha is 0.05, power is 0.80 (beta = 0.20) and an increase in ETPp of 20% in patients compared to controls (mean control group: 321 nmol^*^min/L; mean MM patients: 385 nmol^*^min/L; standard deviation: 67.9 nmol^*^min/L) (12), we calculated that we needed at least 18 patients and 18 controls for our study.

## Results

### Baseline Characteristics

Baseline characteristics of patients and controls are summarized in Table 1. We included 21 MM patients and 21 controls in this study with a comparable age and sex distribution. White blood cell count, RBC count, platelet count, hematocrit and hemoglobin level were decreased, and the immature reticulocyte fraction (IFR), red cell distribution width (RDW) and mean platelet volume (MPV) were increased in MM patients compared to controls. In total, 11 patients and 1 control had anemia (hemoglobin lower than 8.7 mmol/L for men and 7.6 mmol/L for women) and 6 patients were thrombocytopenic (platelet count lower than 100^*^10^9^/L). M protein level was lower than 10 g/L for most patients (19 of 21), and lower than 15 g/L in all MM patients.

**Table 1:** Baseline characteristics of patients and controls.

	**All (*n =* 42)**	**Patient (*n =* 21)**	**Control (*n =* 21)**	***P*-values**
Age, years	67 (53–74)	66 (57–75)	67 (51–73)	ns
Female [*n* (%)]	18 (43%)	8 (38%)	10 (48%)	ns
***Cell count***				
White blood cell count, *10^9^/L	5.8 (4.2–7.2)	4.5 (4.0–6.9)	6.2 (5.3–7.5)	0.03
Red blood cell count, *10^12^/L	4.5 (3.9–5)	4.0 (3.4–4.5)	4.8 (4.4–5.4)	0.001
Hemoglobin, mmol/L	8.8 (7.9–9.4)	8.1 (6.7–9.1)	9.0 (8.4–9.5)	0.014
Haematocrit, %	41.9 (38.7–44.9)	39.6 (32.6–44.3)	43 (41–46.3)	0.014
Immature reticulocyte fraction, %	12.3 (9.7–17.1)	15.9 (12.9–26.7)	10.1 (8.3–11.6)	<0.001
Red cell distribution width, fL	46 (42.6–55.0)	54.9 (50.3–60.3)	42.6 (40.3–44.8)	<0.001
Mean platelet volume, fL	10.1 (9.6–11)	10.3 (9.8–11.4)	9.8 (9.5–10.5)	0.041
Platelet count, *10^9^/L	222 (156–285)	178 (76–222)	250 (222–292)	<0.001
Platelet distribution width, fL	11.2 (10.2–12.7)	11.6 (10–13.6)	10.7 (10.1–11.9)	ns
Anemia [*n* (%)]	12 (28.6%)	11 (52.4%)	1 (4.8%)	
Thrombocytopenia [*n* (%)]	6 (14.3%)	6 (28.6%)	0 (0%)	
***Medication** [n (%)]*		
Platelet inhibitor	13 (31%)	11 (52.4%)	2 (9.5%)	
Anticoagulants	6 (14.3%)	4 (19%)	2 (9.5%)	
Immunomodulatory imide drugs	14 (33.3%)	14 (66.7%)	0	
Corticosteroid	11 (26.2%)	11 (52.4%)	0	
Proteasome inhibitor	5 (11.9%)	5 (23.8%)	0	
Antibiotics	11 (26.2%)	11 (52.4%)	0	
Laxation	8 (19%)	8 (38.1%)	0	
Gastric acid inhibitor	9 (21.4%)	9 (42.9%)	0	
Ca+Vit D3	5 (11.9%)	4 (19%)	1 (4.8%)	
Antivirus	7 (16.7%)	7 (33.3%)	0	
Antihypertension	10 (23.8%)	7 (33.3%)	3 (14.3%)	
Asthma medication	4 (9.5%)	4 (19%)	0	
Uric acid inhibitor	5 (11.9%)	5 (23.8%)	0	
Cholesterol lowering drug	6 (14.3%)	4 (19%)	2 (9.5%)	

*Median and Interquartile ranges (25–75%) or percentage (%) are indicated. Mann-Whitney U test was used for comparison*.

### Thrombin Generation Profile in MM

**Figure 1:**
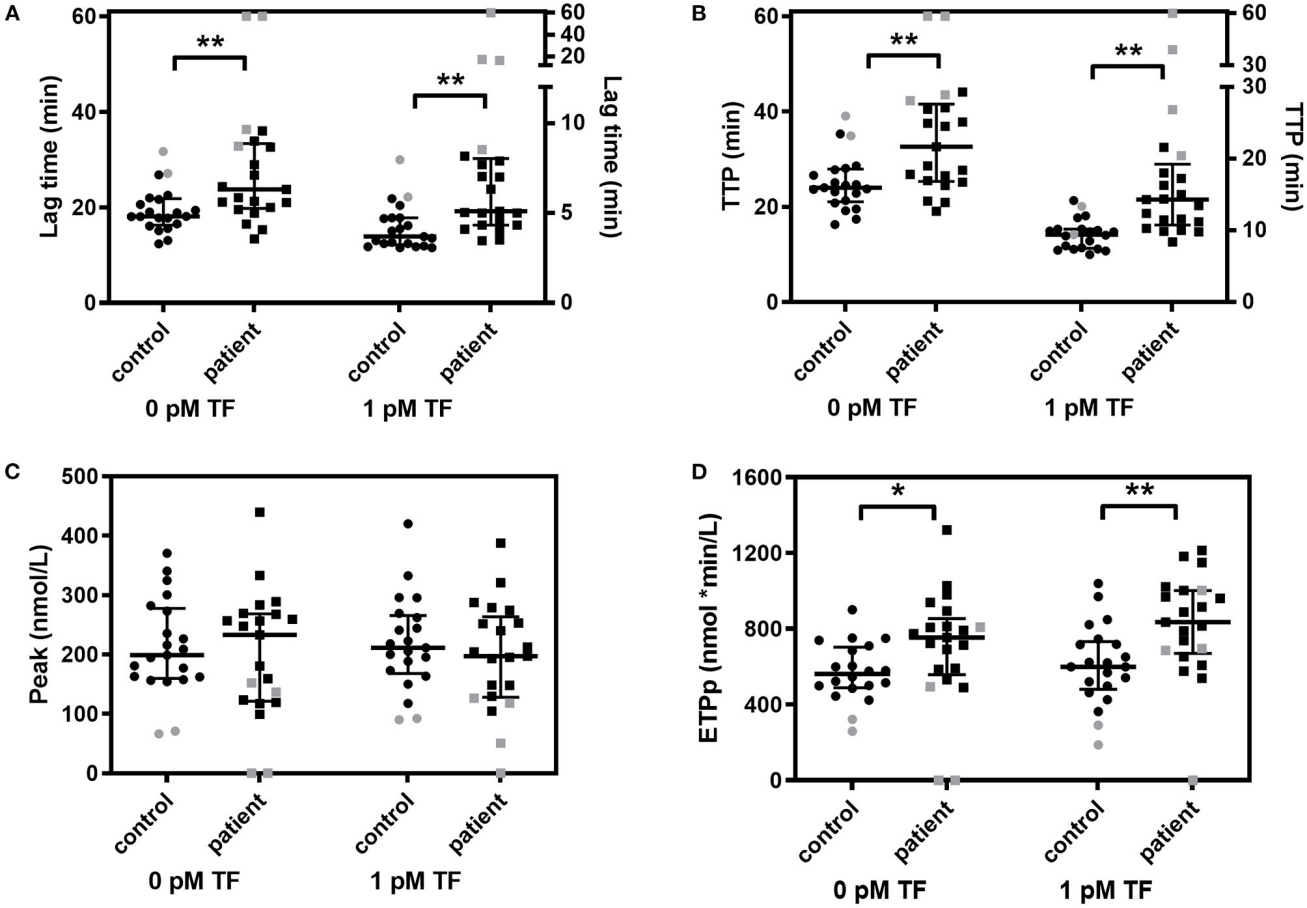
Whole blood thrombin generation in multiple myeloma (MM) patients and controls. Thrombin generation was stimulated with 0 pM and 1 pM TF in whole blood of MM patients and controls. **(A)** Lag time, **(B)** peak thrombin, **(C)** time-to-peak (TTP) and **(D)** endogenous thrombin potential until the peak (ETPp) are presented. Median and IQR are presented as error bars. Patients and controls on anticoagulant medication are indicated in gray. *P < 0.05, **P < 0.01.

To study the effect of MM on coagulation and specifically determine the contribution of blood cells, we measured thrombin generation in PPP (Table 2) and in whole blood (Table 2 and Figure 1) of MM patients and controls. In total, 4 MM patients received anti-coagulant therapy (1 patient received vitamin K antagonist, 1 patient received low molecular weight heparin and two patients received Xarelto) and 2 controls (both received vitamin K antagonists).

**Table 2:** Plasma thrombin generation profiles, whole blood generation profiles and whole blood platelet activation profiles of the study subjects.

	**Patients**	**Controls**	***p*-value**
**Plasma thrombin generation**	*N* = 21	*N* = 21	
*Lag time (min)*			
0 pM TF	16 (13–19)	16 (15–22)	0.237
1 pM TF	6 (5–8)	5 (5–7)	0.84
5 pM TF	3 (3–4)	3 (3–3)	0.504
*TTP (min)*			
0 pM TF	18 (16–23)	20 (18–25)	0.385
1 pM TF	10 (9–12)	11 (9–12)	0.399
5 pM TF	6 (6–8)	7 (6–8)	0.268
*Peak (% of NPP)*			
0 pM TF	146 (107–225)	154 (98–183)	0.554
1 pM TF	193 (127–268)	161 (132–204)	0.252
5 pM TF	126 (95–159)	108 (87–125)	0.187
*ETPp (% of NPP)*			
0 pM TF	140 (105–171)	129 (114–148)	0.554
1 pM TF	151 (120–187)	140 (131–159)	0.642
5 pM TF	112 (94–136)	108 (94–126)	0.87
**Whole blood thrombin generation**	N = 21	N = 21	
*Lag time (min)*			
0 pM TF	24 (20–33)	18 (16–22)	0.005
1 pM TF	5 (4–8)	4 (3–5)	0.001
*TTP (min)*			
0 pM TF	33 (25–42)	24 (21–28)	0.003
1 pM TF	14 (11–19)	9 (8–10)	<0.001
*Peak (nmol/L)*			
0 pM TF	233 (121–269)	199 (160–278)	0.66
1 pM TF	197 (128–264)	211 (168–266)	0.473
ETPp (nmol * min/L)			
0 pM TF	754 (558–854)	577 (492–725)	0.038
1 pM TF	835 (670–1002)	600 (481–732)	0.004
**Whole blood platelet activation**	*N* = 20	*N* = 20	
*αIIbβ3 activation (MFI)*			
Baseline	105 (92–149)	115 (104–138)	0.192
TRAP	151 (107–262)	467 (272–561)	<0.001
MeSADP	1,363 (986–1,963)	2,254 (1,354–3,239)	0.018
CRP-XL	3,253 (2,510–4,069)	4,429 (3,329–4,840)	0.008
*P-selectin expression (MFI)*			
Baseline	90 (84–112)	89 (86–113)	0.883
TRAP	5,842 (5,434–7,014)	7,124 (6,306–7,897)	0.03
MeSADP	2,314 (1,190–2,586)	2,383 (1,052–5,071)	0.512
CRP-XL	6,321 (5,480–7,261)	6,554 (6,085–8,060)	0.242
Platelet-monocyte complexes (%)	6.55% (5.1–11.675%)	6.25% (4.25–9.125%)	0.273

*Median and Interquartile ranges (25–75%) are indicated. The Mann-Whitney U test was used to compare between groups. CRP, collagen related peptide; ETPp, endogenous thrombin potential until the peak; MFI, Median fluorescent intensity; NPP, normal pooled plasma; TF, tissue factor; TRAP, thrombin receptor activating peptide; TTP, time to peak*.

The global plasma coagulation profile did not differ between MM patients and controls (Table 2). None of the TG parameters were different between patients and controls, indicating that coagulation of MM patients was not affected by a factor in plasma.

The coagulation profile in WB was different between MM patients and controls on several aspects (Table 2 and Figure 1). Patients with MM had a longer lag time and TTP, upon stimulation with 0 pM or 1 pM TF. The median lag time was increased up to 38% and the TTP was up to 52% increased in patients compared to controls. The WB-TG ETPp was higher in MM patients than in controls when TG was stimulated with 1 pM TF, the median ETPp was 39% higher in patients compared to controls. Remarkably, the peak amount of thrombin formed was comparable between MM patients and controls. To exclude that the observed difference between MM patients and controls was caused by the use of anticoagulants, we also performed the analysis in the absence of patients and controls on anticoagulants (see Supplementary Table 1). The difference in WB-TG parameters between patients and controls remained.

### Effect of Medication on WB-TG Profile

Patients were classified in groups according to their medication usage (anticoagulants, platelet inhibitor, IMiDs, corticosteroid or proteasome inhibitor) (see Supplementary Table 2). The lag time and ETPp of patients, regardless of the medication used, remained increased in patients with MM compared to controls. Only the use of anticoagulants significantly impacted WB-TG, for the other drugs the WB-TG parameters were not significantly different between patients that did or did not receive a certain drug.

### Contribution of Blood Cells to WB-TG

Results from WB-TG showed that blood cells are important contributors to the coagulation profile in MM patients. Therefore, platelet function was studied by measuring the platelet response to different activation triggers (5 μg/mL CRP-XL, 30 μmol/L TRAP and 2 μmol/L MeSADP) using flow cytometry in 20 MM patients and 20 controls (Table 2). Irrespective of the agonist used, αIIbβ3 activation was lower in MM patients than in controls. P-selectin expression in response to TRAP was also lower in patients compared to controls, while the MeSADP and CRP-XL induced P-selectin expression did not differ between the two groups. The baseline platelet activation level and the platelet-monocyte complexes (PMC) were comparable between MM patients and controls.

**Figure 2:**
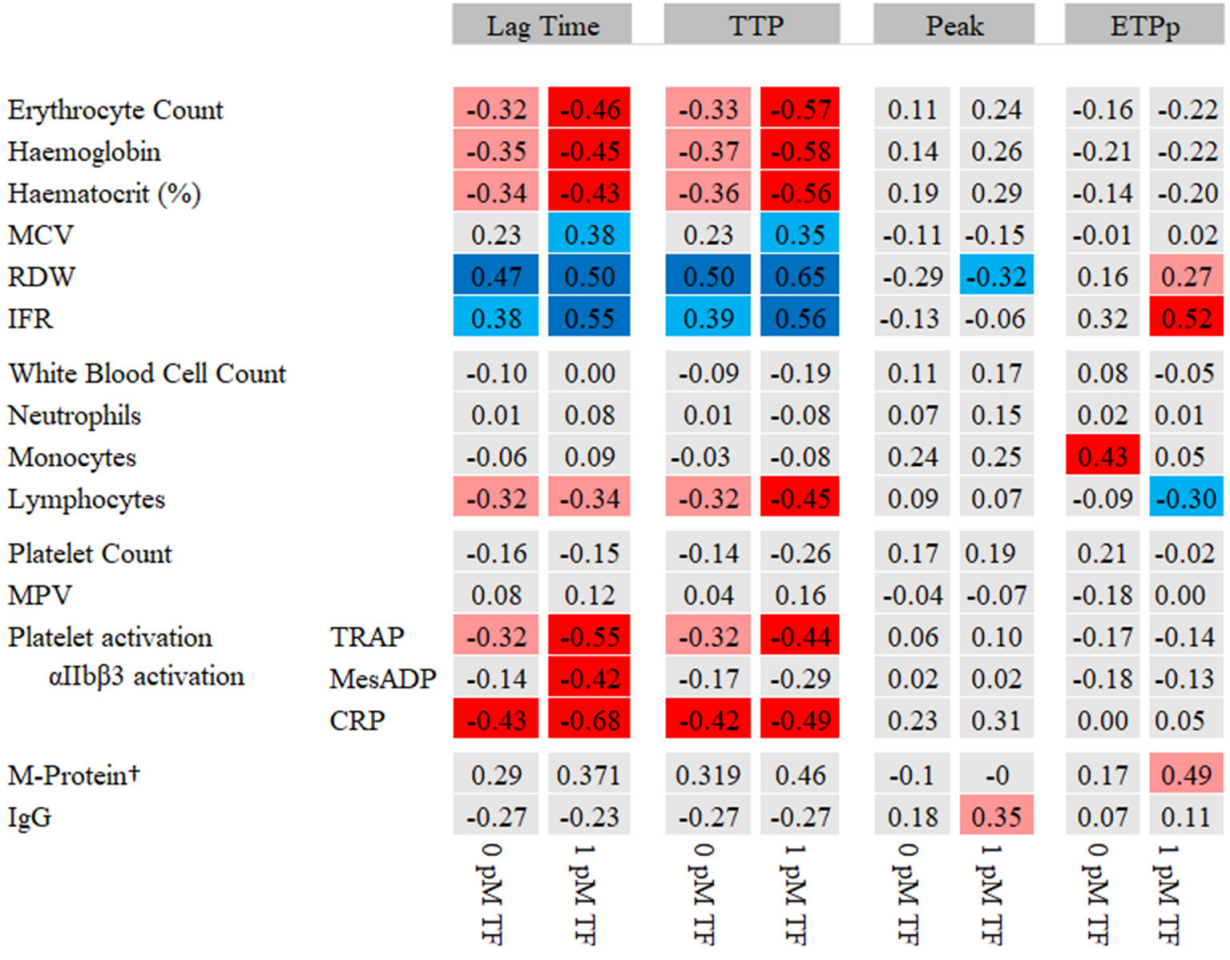
Heat map of the correlation between WB-TG parameters and blood cell parameters. Spearman correlation statistics are shown. In red, a negative correlation with lag time and TTP and a positive correlation with peak and ETPp are presented. In blue, a positive correlation with lag time and TTP and a negative correlation with peak and ETPp are presented. *P* < 0.05 depicted in light red/light blue. *P* < 0.01 depicted in dark red/dark blue. †M-protein levels were only determined in patients. CRP, collagen related peptide; ETPp, endogenous thrombin potential until the peak; IFR, immature reticulocyte fraction; MCH, mean corpuscular hemoglobin; MCV, mean corpuscular volume; MPV, mean platelet volume; RDW; red cell distribution width; TRAP, thrombin receptor activating peptide; TTP, time to peak.

To gain insights into the relation between blood cell fractions and WB-TG in MM patients and controls, we tested the association between WB-TG parameters and the blood cell parameters and platelet activation markers (Figure 2). The lag time and TTP were negatively correlated with RBC count, hemoglobin, hematocrit, lymphocyte count and platelet activation makers and positively correlated with RBC indices (RDW and IFR). Peak and ETPp correlated less with blood cell parameters and did not correlate with platelet activation markers.

## Discussion

The current study shows that patients with MM have an imbalanced hemostasis. On one hand the ETPp in whole blood is increased, which indicates a hypercoagulable state, while on the other hand the lag time and the TTP in WB-TG are prolonged. This imbalanced hemostasis may explain the paradox that MM patients have a high incidence of venous thromboembolism and a high prevalence of bleeding symptoms (1, 2, 22, 23). Additionally, we observed lower platelet numbers, impaired platelet function and lower RBC count in MM-patients, which may further explain the high prevalence of bleeding complications in MM. It is our hypothesis that platelets and RBCs play a crucial role in the balance between a pro- and anti-coagulant phenotype in MM patients.

The pro-coagulant phenotype of MM patients may depend on patient- and disease-related factors and on (side-)effects of therapy (24). We did not find a strong effect of medication on WB-TG profiles, although the numbers of patients in this sub-group analysis does not allow to draw firm conclusions.

Previous studies observed increased plasma levels of FVIII and VWF, increased activated protein C (APC) resistance and reduced soluble thrombomodulin levels in MM patients (25). Furthermore, patients with MM have more phosphatidylserine (PS) positive blood cells (26) and hyperactivation of platelets (11, 27). Our study confirms that blood cells, especially RBCs and platelets, are crucial players in the disbalanced hemostasis in MM. We showed that TG in the absence of blood cells (PPP) was comparable between patients and controls, whereas in WB the TG parameters were indicative for both a hypo- and a hypercoagulable state. Furthermore, MM patients had a significant lower platelet and RBC counts than controls and the response of platelets to platelet agonists was reduced. We showed that platelet function, RBC numbers, RBC turnover and RBC distribution were associated with WB-TG parameters, indicating that platelets and red blood cells regulate the coagulation phenotype. We did not find a relation between white blood cells and thrombosis phenotype in MM-patients, although we think that this should be further studied with more dedicated TG-tests. Remarkably, our findings that WB-TG lag time was prolonged in MM patients, suggest that TF on microparticles or on white blood cells, as was previously observed (28), does not explain our findings of an imbalanced coagulation profile in MM patients.

The recent improvements of the WB-TG approach is a major strength of our study, which allowed us to explore coagulation in MM-patients in the presence of blood cells (12). Several studies have tried to study coagulation in MM-patients with standard plasma thrombin generation profiles. The results of those studies were inconclusive: some studies observed an increased TG (ETP) in PPP (29–32), other studies, including ours, found no difference between MM patients and controls (33, 34) while there are also studies that reported a reduced TG (ETP) in MM patients (35, 36). One study observed no effect on the ETP and Peak, however, the TTP was reduced and the velocity index increased in MM patients compared to controls (37). Altogether, there is no consistent evidence that plasma of MM patients was pro-coagulant, implying that the pro-coagulant phenotype of MM-patients may be a consequence of the blood cell components.

In line with our observations, another study investigating the role of autologous platelets in TG found that MM patients are pro-coagulant (32). Using PRP-TG, it was shown that ETP was higher in MM patients than in controls, while Lenalidomide treatment further increased the ETP in PRP-TG. The lag time nor TTP were reported in this study (32). Our observation that ETPp in MM patients was increased in WB-TG confirms this finding. Furthermore, we observed that the lag time and the TTP in a WB-TG setting were prolonged in MM-patients. This fragile balance between anti- and pro- coagulant phenotype is in line with the clinical knowledge that MM patients have both a high bleeding risk and a high thrombosis incidence (2, 24, 38).

Our findings that 29% of the MM patients were thrombocytopenic, while most of the patients had a reduced agonist induced platelet response, a prolonged lag time and an increased TTP, is in line with clinical knowledge that MM patients have an increased incidence of hemorrhage (38). Platelet dysfunction problems have been previously reported in patients with MM (39) and in patients with hematologic cancers (40). Moreover, it has been suggested that M protein produced by the malignant plasma cells may also directly affect coagulation. In our study, even though all patients received adequate treatment to stabilize M protein levels below 15 g/L, M-protein levels were still positively correlated with ETPp in WB-TG stimulated with 1 pM TF.

RBCs are the most abundant cells in blood, accounting for approximately 35–45% of the blood volume. The current observation that RBC count and high RBC turnover indices were associated with a shorter lag time and TTP is in line with the notion that RBCs promote thrombin generation *in vitro* (10, 41). This was further confirmed by experiments on RBC reconstitution to healthy plasma, which showed that thrombin generation profiles are dose-dependently dependent on RBC count (10, 12). Furthermore, it has been shown that RBC distribution width is associated with higher risk of venous thrombosis and arterial thromboembolism (42, 43), which supports our findings of a correlation between the red cell distribution width and ETPp and peak height in WB-TG.

A major strength of the current study is the inclusion of partners, or close friends of the MM patients as control group, because this ensured comparable distribution for gender, age, ethnic background, lifestyle and social and economic state. Furthermore, patients and their relatives were invited together, to ensure identical pre-analytical treatment of blood at the same time, in the same room, by the same technician.

The most important limitation of our study is the small sample size. Although our study has sufficient power to show that WB-TG parameters were different in MM-patients and controls, our population size does not allow in-depth subgroup analysis to link WB-TG to treatment strategy. Another limitation is the case control design, which cannot be used to predict disease outcome in MM patients, because we do not have follow-up data.

In summary, we have shown that MM patients have a slower onset of thrombin formation in whole blood, while they have an increased thrombin formation capacity. MM patients seem to balance on a thin line between a pro- and an anti-coagulant phenotype and only minimal triggers can push patients to either thrombotic complications or to bleeding events. The TG in PPP was not different from controls, indicating that the blood cells, but not the plasma, is responsible for the disbalanced hemostatic phenotype of MM-patients. Disbalanced hemostasis may be an effect of disease characteristics, but it may also be a side effect of the intensive treatment of patients. The next logical step will be a large cohort study, which determines the WB coagulation phenotype at baseline to follow the patients on bleeding and VTE incidence during follow up.

## Data Availability Statement

The raw data supporting the conclusions of this article will be made available by the authors, without undue reservation.

## Ethics Statement

The studies involving human participants were reviewed and approved by Medical Ethical Committee of Maastricht University Medical Center. The patients/participants provided their written informed consent to participate in this study.

## Author Contributions

JK and MR designed the research. RF and KS recruited the patients. LL, MR, and YS performed experiments and collected the data. YS, MR, JW, and JK performed data analysis. LL, MR, and JK wrote the original manuscript. LL, MR, YS, JR, RF, KS, DH, JW, BL, and JK revised the manuscript. JK, MR, and JR supervised the study. All authors contributed to the article and approved the submitted version.

## Funding

LL, YS, and JW report grants from China Scholarship Council (File Nos. 201706230245, 201606790009, and 201606130068) during the conduct of the study.

## Conflict of Interest

The authors declare that the research was conducted in the absence of any commercial or financial relationships that could be construed as a potential conflict of interest.

## Publisher's Note

All claims expressed in this article are solely those of the authors and do not necessarily represent those of their affiliated organizations, or those of the publisher, the editors and the reviewers. Any product that may be evaluated in this article, or claim that may be made by its manufacturer, is not guaranteed or endorsed by the publisher.
