# Supplementary material for: Patients With Multiple Myeloma Have a Disbalanced Whole Blood Thrombin Generation Profile
Source: Front Cardiovasc Med. 2022 Jun 27;9:919495. doi: 10.3389/fcvm.2022.919495 (PMC9271700; doi:10.3389/fcvm.2022.919495)
Supplement: Supplementary file 1 [file Table_1.pdf]

Supplementary Material

Supplemental table 1. Whole blood generation profile of multiple myeloma patients and controls

	All patients and controls			Patients and controls on anticoagulants excluded		
	Patients	Controls	p-value	Patients	Controls	p-value
	N = 21	N = 21		N = 17	N = 19	
Lag time (min)						
0 pM TF	24 (20-33)	18 (16-22)	0.005	21 (19 - 28)	18 (16 - 21)	0.011
1 pM TF	5 (4-8)	4 (3-5)	0.001	5 (4 - 7)	4 (3 - 5)	0.001
TTP (min)						
0 pM TF	33 (25-42)	24 (21-28)	0.003	25 (19 - 28)	21 (16 - 24)	0.008
1 pM TF	14 (11-19)	9 (8-10)	<0.001	12 (10 - 16)	9 (7 - 10)	<0.001
Peak (nmol/L)						
0 pM TF	233 (121-269)	199 (160-278)	0.66	257 (142 - 277)	209 (163 - 282)	0.918
1 pM TF	197 (128-264)	211 (168-266)	0.473	213 (171 - 277)	218 (188 - 269)	0.77
*ETPp (nmol *min/L)*						
0 pM TF	754 (558-854)	577 (492-725)	0.038	775 (642 - 917)	578 (500 - 718)	0.002
1 pM TF	835 (670-1002)	600 (481-732)	0.004	889 (696 - 1012)	621 (521 - 747)	0.003

Median and Interquartile ranges (25%-75%) are indicated. The Mann-Whitney U test was used to compare between groups. The columns on the left show the results for all patients and controls, the columns on the right show the results after exclusion of patients and controls on anticoagulants.

ETPp, endogenous thrombin potential until the peak; TF, tissue factor; TTP, time to peak.

Supplemental table 2. The effect of medication on whole blood thrombin generation in multiple myeloma patients and controls.

	Control	Patients without medication	Patients with medication	p-value
Anticoagulants (n)	21	17	4	
Lag time (min)	3.7 (3.2 - 4.7)	5.0 (4.2 - 7.0)	17.0 (10.5 - 49.4) *	0.0003
ETPp (nmol*min/L)	600 (481 - 732)	915 (721 - 1012)	691 (172 - 728)	0.048
Platelet inhibitor (n)	21	10	11	
Lag time (min)	3.7 (3.2 - 4.7)	6.0 (4.8 - 16.8)	5.1 (4.0 - 7.9)	0.27
ETPp (nmol*min/L)	600 (481 - 732)	765 (642 - 923)	968 (738 - 1022)	0.25
Immunomodulatory drugs (n)	21	7	14	
Lag time (min)	3.7 (3.2 - 4.7)	5.0 (4.6 - 8.2)	6.7 (4.1 - 8.1)	0.54
ETPp (nmol*min/L)	600 (481 - 732)	889 (789 - 961)	777 (642 - 1054)	0.93
Corticosteroids (n)	21	10	11	
Lag time (min)	3.7 (3.2 - 4.7)	4.5 (3.9 - 9.4)	7.0 (5.0 - 8.2)	0.13
ETPp (nmol*min/L)	600 (481 - 732)	743 (634 - 974)	961 (738 - 1002)	0.28
Proteasome inhibitor (n)	21	16	5	
Lag time (min)	3.7 (3.2 - 4.7)	6.7 (4.4 - 8.4)	5.0 (4.2 - 6.5)	0.43
ETPp (nmol*min/L)	600 (481 - 732)	764 (619 - 992)	968 (902 - 1118)	0.05

Thrombin generation was stimulated with 1 pM TF in whole blood of MM patients and controls. The effect of medication on the lag time and endogenous thrombin potential until the peak (ETPp) are shown in the table. Median and Interquartile ranges (25%-75%) are indicated. The difference in WB-TG parameters between patients on the specified medication were compared with those who did not use this specific medication (Mann-Whitney U statistical test) * Patients that used anticoagulants had a prolonged lag time and reduced ETPp compared to patients that did not take anticoagulants. None of the other tested treatments reached statistical significance between users and non-users.
